# Visceral Mobilization and Functional Constipation in Stroke Survivors: A Randomized, Controlled, Double-Blind, Clinical Trial

**DOI:** 10.7759/cureus.8058

**Published:** 2020-05-11

**Authors:** Hugo Pasin Neto, Rodolfo A Borges

**Affiliations:** 1 Osteopathy, Brazilian College of Osteopathy, Sorocaba, BRA; 2 Physiotherapy, University of Sorocaba, Sorocaba, BRA

**Keywords:** stroke, visceral mobilization, osteopathic manipulative treatment, functional constipation, balance

## Abstract

Introduction

Chronic functional constipation is common among stroke survivors. Osteopathy is an effective form of treatment as it acts on the structures surrounding the bowels that may have lost their normal capacity of resilience. The aim of the present study was to evaluate the effect of visceral mobilization on symptoms of functional constipation and static balance in stroke survivors.

Materials and methods

Thirty stroke survivors met the eligibility criteria and were randomly allocated to a group physical therapy and visceral manipulation or a group physical therapy. Both groups were submitted to conventional physical therapy. The group physical therapy and visceral manipulation was also submitted to visceral mobilization (sphincter inhibition and mobilization of the large intestine), whereas the group physical therapy was submitted to a sham procedure (superficial touching over the intestines). Evaluations were conducted prior to the intervention, immediately after the first intervention session and one week after the end of the five sessions. At each evaluation, the static balance was analyzed using a computerized plantar pressure sensor. Moreover, an intestinal symptoms rating scale was administered during the pre-intervention evaluation, and one week after the end of the intervention.

Results

Significant improvements were found in intestinal symptoms (frequency of bowel movements, abdominal pain/discomfort, difficulty eliminating stools, sensation of intestinal swelling or distention, difficulty eliminating gas, sensation of incomplete bowel movement and, anal pain during bowel movement) and static balance (anteroposterior sway: F = 82.06, p = 0.0001; velocity of anteroposterior sway: F = 17.6, p = 0.001; and velocity of mediolateral sway: F = 4.41, p = 0.01).

Conclusion

Visceral mobilization can be part of a neurologic rehabilitation program to improve symptoms of constipation and static balance in stroke survivors.

## Introduction

Functional constipation is an intestinal motility disorder that is highly prevalent throughout the world [1]. According to the Rome III Consensus, this condition is defined by two or more of the following criteria in a six-month period: fewer than three bowel movements per week, straining during defecation, hardened or fragmented stools, sensation of anorectal obstruction/blockage and the use of manual maneuvers to facilitate bowel movement [2,3].

Previous studies report that the prevalence rate of functional constipation ranges from 2.6% to 30.7% and the condition is more common in females and seniors [3]. In agreement with these data, Schmidt et al. conducted an epidemiological study involving the Brazilian population and found a 25.2% rate of functional constipation among the individuals interviewed, with greater occurrences in the female gender and elderly population [3].

Diseases that affect the central nervous system play an important role in the development of functional constipation, among which cerebrovascular accident (stroke) seems to have the strongest association with this condition [3]. Indeed, intestinal symptoms are very common in stroke survivors. The prevalence of functional constipation in this population ranges from 50% in the first month following a stroke to 30% after a mean of 36 weeks [4-6]. Moreover, this complication occurs independently of the side of the brain affected [7].

The causes of constipation include immobility, insufficient water intake, lowered consciousness, abnormal colon contractibility, and the side effects of medications. Among stroke survivors, this disorder has been explained by neurological impairment, dependence and a prolonged hospital stay as well as motor, cognitive and communicative impairments [3-5]. Moreover, studies have demonstrated that colon transit is significantly reduced in stroke survivors, which Mach attributes to the dysregulation of the central nervous system combined with abnormal passive movement of the visceral organs caused by limitations in body movements [3,8].

According to Bassotti et al., symptoms such as abdominal distension, a sensation of abdominal fullness, continuous or sharp pain, psychological discomfort and pain/discomfort in segments of the trunk compromise the quality of life of individuals with constipation [9].

Another aspect to consider is that stroke survivors with hemiparesis have muscle weakness and impaired control on the affected side of the body, with a reduction in range of motion as well as the occurrence of pain, which can lead to changes in the center of pressure of the sole of the foot, thereby affecting static balance [10].

The findings of previous studies suggest that treatments for chronic constipation are expensive, often invasive and not always effective, especially in the long term [11]. The most common form of treatment involves changes in living habits, such as the ingestion of water and dietary fiber, and for those in whom the problem is not solved with these measures, the alternatives include the use of laxatives, biofeedback, enema or surgery [12].

Osteopathy is a diagnostic and treatment method based on the principles of the unity of the body and involves structural, cranial, and visceral approaches. The visceral approach consists of a set of manual techniques used to diagnose and normalize mechanical, vascular, and neurological dysfunctions of the bowels and improve their functioning [13]. According to Hundscheid et al. the osteopathic treatment of constipation is effective because the structures surrounding the peritoneal bowels may have lost their normal capacity of resilience [14]. Thus, the goal of osteopathy is to restore the movement of abdominal organs and reestablish the functional characteristics of the tissues involved.

The aim of the present study was to investigate the effect of mobilization of the intestine on signs and symptoms of functional constipation and static balance in stroke survivors.

## Materials and methods

Study design

A prospective, analytical, paired, randomized, controlled, double-blind, longitudinal, clinical trial was conducted.

Setting

This project was developed at the Integrated Movement Analysis Lab of University Nove de Julho (São Paulo, SP, Brazil) and the Integrated Human Movement Analysis Lab of the University of Sorocaba (Sorocaba, SP, Brazil) between September 2016 and January 2017. This study is registered with the clinicaltrials.gov - service of the U.S. National Institutes of Health (Registration Number: NCT03031977) and approval from the Human Research Ethics Committee of the institution University of Sorocaba under process number 54042216.2.0000.5500 in compliance with the ethical standards by the Declaration of Helsinki.

Inclusion and exclusion criteria

Male and female patients aged 40 to 70 years having suffered a stroke more than one year earlier with hemiparesis secondary to a single unilateral event, the capacity for independent gait and a complaint of chronic constipation for more than six months in accordance with the definition of functional constipation described by the Rome III Consensus were considered for inclusion in the study [2,3]. The exclusion criteria were an incision or tumor in the abdominal region, fractures, rheumatic disease, infectious process in the acute phase, inability to understand the proposed evaluations and inability to walk or maintain balance in an independent manner.

Procedures

All volunteers received clarifications with regard to the procedures and were informed that the procedures would not affect their health. The volunteers were assured that all information would be confidential and their privacy would remain protected. All volunteers who agreed to participate in the study signed a statement of informed consent in compliance with Resolution 196/96 of the Brazilian National Board of Health.

Sham intervention procedures were always performed in combination with active conventional therapy, which lessened the impact of the sham procedure on the patient. Moreover, the patients were informed of the use of this procedure prior to the onset of the study.

Randomization, evaluation, and intervention

Thirty individuals met the eligibility criteria and were randomly allocated to one of the two study groups using a block randomization method. Fifteen patients were allocated to each group: group physical therapy and visceral manipulation - conventional physical therapy and visceral mobilization; group physical therapy: conventional physical therapy and sham mobilization. Block randomization involved the use of sealed opaque envelopes, each containing a card stipulating one of the two groups. After the pre-intervention evaluation, the participant was allocated to a group by opening an envelope. This process was performed by a member of the research team who was not involved in the recruitment process or development of the study.

Evaluations were performed on three occasions: 1) prior to the intervention, 2) immediately after the first session, and 3) one week after the last session. All specific evaluation procedures were performed during the pre-intervention and post-intervention (one week after the sessions) evaluations, whereas only the computerized plantar pressure evaluation was performed at the second evaluation (immediately after the first session). The researcher in charge of the evaluations was blinded to the objectives of the study and did not take part in the intervention protocols. Moreover, the order of the evaluations was randomized to avoid the effect of standardization.

First, an identification chart was filled out with information on age, sex, date of stroke episode, and onset of constipation. Anthropometric data (body mass and height) were also measured and recorded. The specific evaluation procedures consisted of the use of an intestinal symptoms rating scale (primary outcome) and plantar pressure evaluation (secondary outcome) [15].

The ten-item intestinal symptoms rating scale was used to measure the intensity of intestinal symptoms. On this scale, each item is scored from 0 to 4 points. Item 1 regards the frequency of bowel movements and Items 2 to 10 address intestinal symptoms. The final scores is calculated by the mean of the item scores; 0 corresponds to a absence of symptoms and 4 corresponds to the highest intensity of symptoms [15].

The plantar pressure evaluation was performed using a force plate (S-Plate, Medicapteurs, France) with 1600 sensors and an acquisition frequency of 100 images per second. This force plate measures the distribution of plantar pressure during quiet standing, with quantitative data on anteroposterior and mediolateral sway (cm) as well as the mean sway velocity (cm/s) in these directions. The volunteer stood barefoot on the pressure plate in the standing position with arms alongside the body, gaze fixed on the horizon, lips closed, with the mandible and rest of the body relaxed. Readings were performed for 50 seconds, the 30 intermediate seconds of which were used for analysis. The values were recorded using the program provided by the manufacturer installed on a microcomputer.

**Figure 1:**
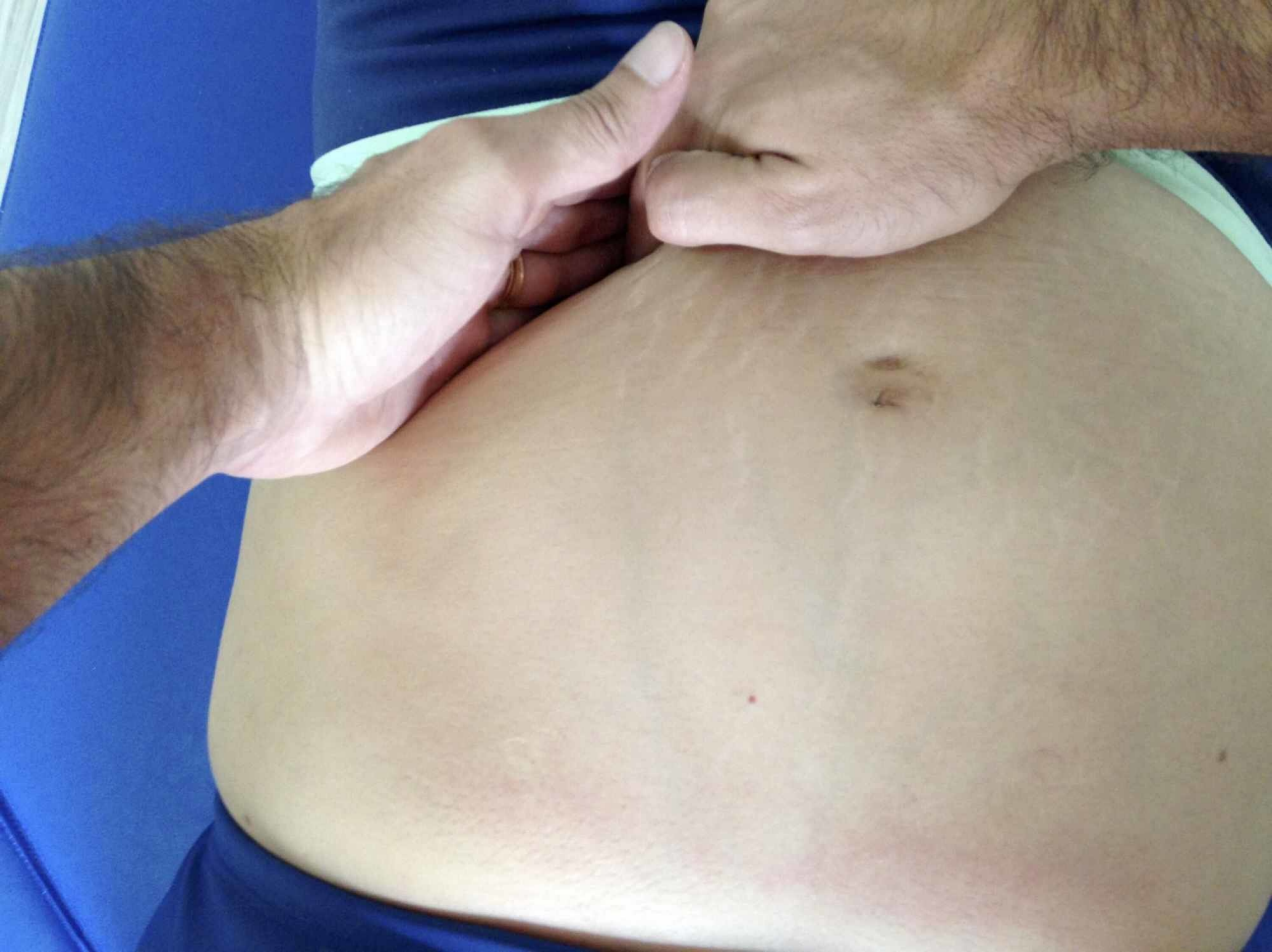
Mobilization of the sigmoid colon

Both groups were submitted to general kinesiotherapy with a focus on strengthening, stretching, and proprioception during conventional physical therapy. The group physical therapy and visceral manipulation was also submitted to mobilization of the ascending colon, descending colon, sigmoid colon, and sphincters (cardiac, pyloric, Oddi, duodenojejunal and ileocecal) with the patient in the supine position, knees flexed, feet supported and abdomen exposed. Contact was made with the region to be treated, leading it in the direction of immobility, with pressure maintained for one minute on each region with intensity based on the sensitivity to tension observed on the feedback of the individual (Figure 1). In the group physical therapy, sham mobilization was performed, which consisted of superficial contact with no pressure on the abdominal region corresponding to the loops of the large intestine. Five intervention sessions were held over a two-week period.

Statistical analysis

The data were first submitted to the Kolmogorov-Smirnov test to determine adherence to the Gaussian curve. The independent t-test was used for the inter-group analysis. Repeated-measures analysis of variance (ANOVA) was used for the intra-group analysis under each condition. The chi-square test was used to evaluate the dispersion of the qualitative variables in the intra-group and inter-group analyses. A p-value ≤ 0.05 was considered indicative of statistical significance. The data were organized and tabulated using the Statistical Package for the Social Sciences, version 19.0 (SPSS Inc., Chicago, USA).

## Results

Table 1 displays the characteristics of the participants. The male gender predominated. The mean age was 66 years and mean time elapsed since the occurrence of stroke was 25 months.

**Table 1:** Characteristics of the sample

	Group physical therapy (n = 15)	Group physical therapy and visceral manipulation (n = 15)	p
Age (years)	68 (9)	63 (5)	0.1
Weight (kg)	67 (7.4)	65 (4.9)	0.4
Height (m)	1.63 (0.11)	1.65 (0.8)	0.8
Time since stroke (months)	26 (7)	21 (5)	0.3
Time with constipation (months)	23 (4)	20 (6)	0.4
Gender	n (%)	n (%)	
Male	11 (73)	13 (86)	0.9
Female	4 (26)	2 (13)	0.8

**Figure 2:**
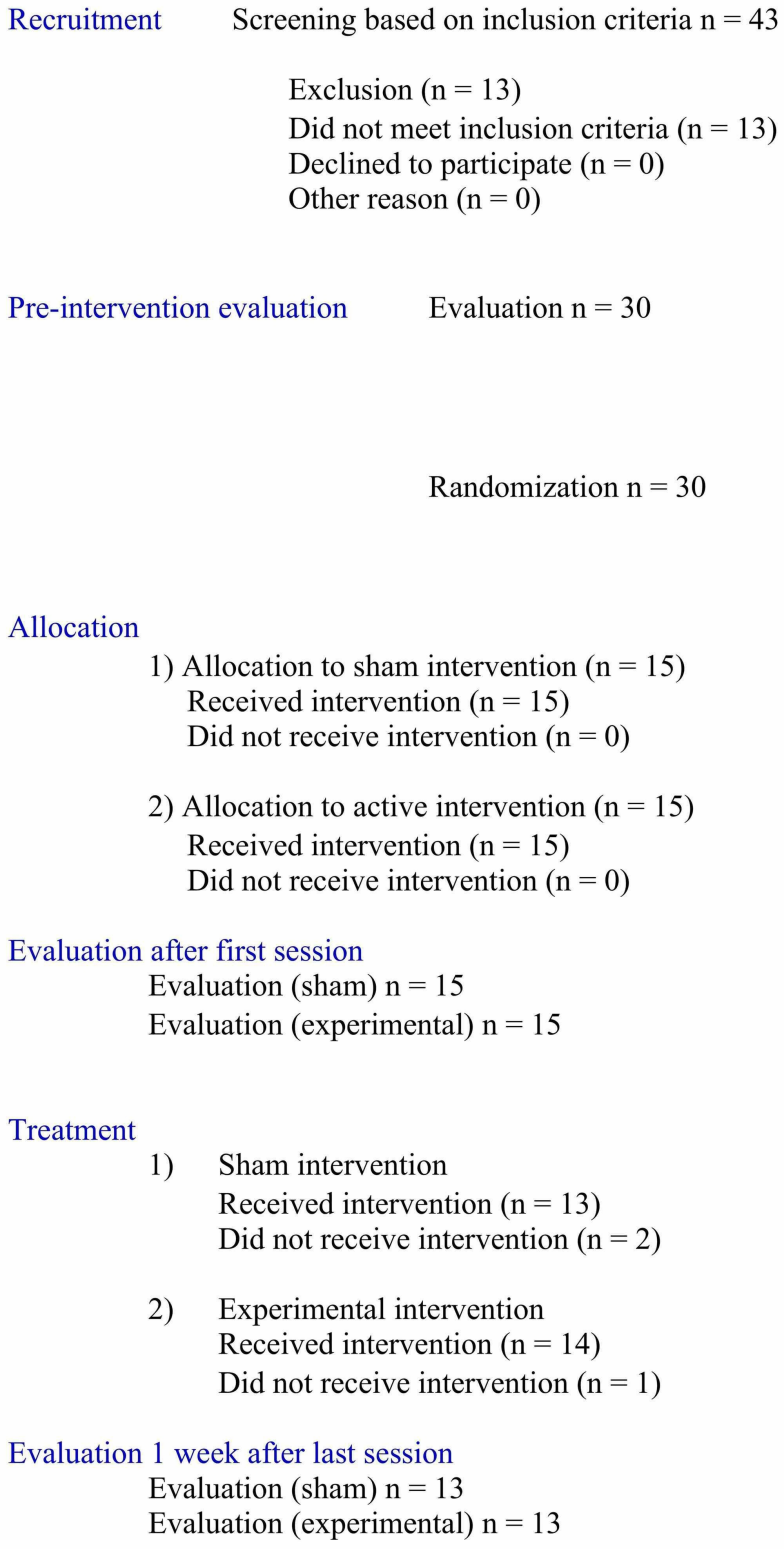
Flowchart

Forty-three individuals were recruited, thirteen of whom were excluded for not meeting the eligibility criteria. The main reason for non-inclusion was a lack of independent gait or balance. Moreover, three volunteers dropped out of the study during the therapy sessions: one in the group physical therapy and visceral manipulation and two in the group physical therapy. Figure 2 shows a detailed flowchart of the recruitment, exclusion, evaluation, and intervention processes.

No significant difference between groups was found regarding the number of bowel movements per week during the pre-intervention evaluation. In the intra-group analyses, statistically, differences were found in the group physical therapy and visceral manipulation between the pre-intervention and post-intervention (one week after the therapy sessions) evaluations for all variables. In contrast, the only significant difference in the group physical therapy regarded the variable “once a day or once every two days”. Table 2 displays the results of the pre-intervention and post-intervention evaluations in both groups. The results in this table were presented at the Fifth Fascia Research Congress.

**Table 2:** Frequency of bowel movements in groups physical therapy and physical therapy and visceral manipulation before and after intervention Post-intervention: one week after last session; *statistical significance level assumed at p < 0.05 (X2 test)

	Group physical therapy		Group physical therapy and visceral manipulation	
	Pre-intervention	Post-intervention	Pre-intervention	Post-intervention
Once every 4 to 7 days	33.3%	30.7%	26.6%	14.2%*
Once every 3 days	46.6%	38.4%	53.3%	21.4%*
Once per day or once every 2 days	13.3%	30.7%*	20%	50%*
Once or twice a day	6.6%	0%	0%	14.2%*

No significant difference between groups was found regarding the qualitative variables related to the prevalence of intestinal symptoms during the pre-intervention evaluation. In the intra-group analyses, statistically, differences were found in the group physical therapy and visceral manipulation between the pre-intervention and post-intervention (one week after the therapy sessions) evaluations for all variables except “soft or watery stools” and “sensation of urgent need to move bowels”, which were respectively marked by only one individual and no individuals at the two evaluation times. It should be stressed that a symptom was considered present when the volunteer marked the item as either “moderate” or “severe”. Table 3 displays the results of the pre-intervention and post-intervention evaluations in both groups.

**Table 3:** Prevalence of intestinal symptoms in groups physical therapy and physical therapy and visceral manipulation before and after intervention Post-intervention: one week after last session; *statistical significance level assumed at p < 0.05 (X2 test)

	Group physical therapy		Group physical therapy and visceral manipulation	
	Pre-intervention	Post-intervention	Pre-intervention	Post-intervention
Abdominal pain/discomfort	53.3%	53.8%	66.6%	14.2%*
Soft or watery stools	13.3%	15.3%	6.6%	7.1%
Very hard stools or unable to eliminate stools	73.3%	69.2%	66.6%	28.5%*
Strain required to move bowels	53.3%	53.8%	66.6%	35.7%*
Sensation of urgent need to move bowels	6.6%	7.6%	0%	0%
Abdominal swelling or distension	26.6%	30.7%	20%	14.2%
Difficulty passing gas or excessive passing of gas	40%	38.4%	53.3%	21.4%*
Sensation of incomplete bowel movement	53.3%	53.8%	66.6%	28.5%*
Anal pain at time of moving bowels	33.3%	38.4%	46.6%	14.2%*

A statistically significant intra-group difference was found in the group physical therapy and visceral manipulation regarding the intensity of intestinal symptoms based on the mean score, as proposed for the intestinal symptoms scale [pre-intervention: 2.8 (1.08); post-intervention: 1.5 (0.74); (p = 0.04)]. The same did not occur in the group physical therapy, for which the means were practically the same during the two evaluations [pre-intervention: 2.3 (1.1); post-intervention: 2.4 (2.3); (p = 0.83)].

No statistically significant differences between groups were found regarding any of the variables related to plantar pressure at the pre-intervention evaluation. In the intra-group analyses, repeated-measures ANOVA revealed statistically significant differences among the three evaluation times (pre-intervention, after first session and post-intervention) in the group physical therapy and visceral manipulation with regard to anteroposterior sway (F = 82.06; p = 0.0001), velocity of anteroposterior sway (F = 17.6; p = 0.001) and velocity of mediolateral sway (F = 4.41; p = 0.01). The same did not occur with regard to mediolateral sway (F = 0.08; p = 0.92). Table 4 displays the mean and standard deviation values of the variables analyzed during the three evaluations of static balance in the two groups.

**Table 4:** Results of balance analysis in groups physical therapy and physical therapy and visceral manipulation at three evaluation times Evaluation 1: pre-intervention; Evaluation 2: immediately after first therapy session; Evaluation 3: one week after fifth therapy session; *statistical significance level assumed at p < 0.05 (ANOVA test)

	Group physical therapy			Group physical therapy and visceral manipulation		
	Evaluation 1	Evaluation 2	Evaluation 3	Evaluation 1	Evaluation 2	Evaluation 3
Anteroposterior sway (cm)	17.43(5.1)	16.39(7.3)	16.34(5.2)	16.38(6.5)	23.3(8.2)*	12.14(5.1)*
Mediolateral sway (cm)	14.29(7.8)	14.13(9.8)	16.23(8.9)	15.71(8.0)	17.97(9.6)	14.08(11.2)
Velocity of anteroposterior sway (cm/s)	20.13(23.6)	22.45(30.8)	20.9(23.9)	21.04(31.1)	25.44(16.7)*	22.39(21.1)*
Velocity of mediolateral sway (cm/s)	17.44(25)	17.49(24)	18.09(19.9)	17.44(25)	24.84(12.2)*	18(19.9)*

## Discussion

This is the first randomized, controlled, double-blind, clinical trial to evaluate the effects of visceral mobilization on the symptoms of chronic functional constipation in stroke survivors. The results demonstrate improvements in the frequency of bowel movements, abdominal pain/discomfort, difficulty defecating, the sensation of abdominal swelling or distension, difficulty eliminating gas, the sensation of incomplete bowel movement and anal pain during defecation. Thus, visceral mobilization can be included as part of the rehabilitation program for these patients.

Although the literature on this subject is scarce, the present findings are in agreement with data described in some previous studies that demonstrate functional improvement following visceral mobilization, especially in terms of regulating bowel movements in individuals with constipation. In a pilot study involving 13 children with chronic, non-progressive encephalopathy and a diagnosis of chronic constipation, Tarsuslu et al. used a protocol involving the inhibition of the iliopsoas muscles and sphincter combined with intestinal mobilization in three weekly sessions for a six-month period and found an increase in the frequency of bowel movements as well as functional improvements in the patients [16]. In another study, Brugman, Fitzgerald and Fryer found a significant improvement in the severity of constipation, colon transit time and quality of life of individuals with chronic constipation using a visceral mobilization protocol conducted in six sessions over a four-week period [17].

Employing a different approach, Attali et al. and Florance et al. proposed an intervention based on dysfunctions found during the treatment for irritable bowel syndrome (IBS), one of the symptoms of which is constipation [18,19]. In the first study, visceral osteopathic treatment was performed on 31 patients and involved a combination of different techniques. At the beginning of each session, a global technique was employed with a mild vibration over the part of the abdomen the patient reported to be the most sensitive, followed by sacrum manipulation to stimulate the parasympathetic pelvic splanchnic nerves. Significant improvements were found in self-reported diarrhea, abdominal distension and abdominal pain [18]. The second study was a randomized, controlled, clinical trial conducted to evaluate the effect of visceral treatment on the symptoms of IBS. The 30 patients were allocated to either a group physical therapy and visceral manipulation or group physical therapy submitted to a sham procedure. The authors report improvements in the severity of symptoms and the quality of life of the patients [19].

Others study with similar aims have achieved similar results that are in agreement with the present findings. Holey and Lawler demonstrated that abdominal massage performed on a female patient with chronic constipation led to improvements in the symptoms related to this condition [20]. Silva and Motta conducted a study involving abdominal massage, abdominal muscle training and respiratory exercises, reporting an improvement in the frequency of bowel movements in children with constipation [21]. In a sample of 60 volunteers, Lamas et al. found that abdominal massage was effective at reducing the severity of gastrointestinal symptoms [22].

One of the hypotheses that may explain the improvement in symptoms of constipation regards the restoration of the capacity of resilience of the structures that surround the peritoneal bowels during visceral mobilization as well as the capacity of manual techniques to stimulate the organism to produce endocannabinoid substances, which modulate intestinal function [14,23,24]

According to Woolf, visceral mobilization may reduce excessive visceral nociceptive inputs, thereby reducing the likelihood of changes in the excitability of the central nervous system as a result of the increase in afferent signals [25]. Different studies have been conducted to demonstrate this relationship between visceral mobility and the excitability of the nervous system. Investigating the paravertebral muscles at the L1 level, McSweeney et al. found an increase in the pressure pain threshold (measured using algometry) following mobilization of the sigmoid colon [26]. The authors of another study found that visceral mobilization techniques were effective with regard to improving or restoring kidney mobility as well as reducing the perception of pain in the short term among individuals with non-specific low back pain [27]. The results demonstrate that capacity of mobilizations to mold afferent signals and the responses to these signals. With regard to the treatment of constipation, the benefits of the attenuation of these responses through the reduction in the excitability of the nervous system are seen in a study conducted by Orhan et al. [28]. The authors employed Kinesio taping and manipulation of the connective tissue of the posterior region of the trunk in children with cerebral palsy and chronic constipation. The authors attribute the significant improvement in symptoms of constipation to the reestablishment of the balance of the autonomic nervous system.

Considering the results of previous studies, there is strong evidence that visceral mobilization in the present investigation led to an improvement in intestinal mobility, a reduction in the adverse effect on the excitability of the nervous system through sensory afference, a rebalance of the autonomic nervous system and consequent improvements in intestinal function and symptoms. With regard to the plantar pressure evaluations, a direct relationship was found between visceral mobilization and static balance, which lends further strength to the hypothesis of a rebalance of the nervous system after the intervention. Although this is the first study to evaluate this relationship in stroke survivors, a previous investigation offers data that corroborate these findings. Tarsuslu et al. employed the osteopathic method on children with cerebral palsy and found a significant reduction in spasticity (evaluated using the modified Ashworth scale) after the intervention [16]. This finding, together with the improvement in balance demonstrated in the present study one week after the last intervention session, suggests that visceral mobilization can enhance function in neurological patients with chronic constipation.

The anatomic aspect is also relevant when one considers the continuity of the fascia. By transferring the change in tension generating by visceral mobilization to other systems, a global effect may be achieved [29]. According to Kuchera, the unity of the organism is one of the basic principles of visceral treatment and physiological homeostasis is the aim of the restoration of organ mobility [30].

Limitations

The main limitations of the present study are related to the sham visceral mobilization procedure, since it was necessary to apply a light touch over the same regions treated with active mobilization, which enabled the possibility of two sources of bias. The first is the suspicion of the patient with regard to the type of intervention being performed and the second is related to the fact that such contact, although subtle, it could be described as therapeutic and therefore does not truly fulfill its placebo function. However, the nature of the intervention made it necessary to assume this shortcoming. Another limitation regards the fact that the interventions were not performed by a single researcher. To diminish possible divergences related to the use of different therapists, the researchers in charge of the interventions underwent a training exercise to maximize the standardization of the procedures. However, the evaluations were performed by a single researcher (first author), who was unaware of the group to which each volunteer had been allocated.

## Conclusions

Considering the incidence of stroke and the complications generated by constipation in this group of patients, visceral mobilization is a safe, noninvasive therapeutic option and can be part of a neurological rehabilitation program to improve symptoms of constipation and static balance in stroke survivors. Moreover, the functional results, albeit small, suggest the possibility that this technique can assist in functional training protocols.
